# A Comprehensive Evaluation and Benchmarking of Convolutional Neural Networks for Melanoma Diagnosis

**DOI:** 10.3390/cancers13174494

**Published:** 2021-09-06

**Authors:** Saeed Alzahrani, Baidaa Al-Bander, Waleed Al-Nuaimy

**Affiliations:** 1 Department of Electrical Engineering and Electronics, University of Liverpool, Liverpool L69 3GJ, UK; wax@liverpool.ac.uk; 2 Department of Computer Engineering, University of Diyala, Baqubah 32010, Iraq; baidaa@uodiyala.edu.iq

**Keywords:** melanoma, convolution neural networks, benchmarking

## Abstract

**Simple Summary:**

Melanoma is the most dangerous type of skin cancer. It grows quickly and has the ability to spread to any organ. This study aims to evaluate and benchmark deep learning models for automatic melanoma diagnosis considering nineteen convolutional neural networks and ten criteria. Multi-Criteria Decision Making methods (MCDMs) are exploited to conduct the benchmarking and subsequently selecting the optimal model considering the predefined criteria. The study findings would help in the model selection, designing quick and reliable diagnostic tools based on image data, and contributing to the development of more accurate and efficient point-of-care diagnostic and detection systems.

**Abstract:**

Melanoma is the most invasive skin cancer with the highest risk of death. While it is a serious skin cancer, it is highly curable if detected early. Melanoma diagnosis is difficult, even for experienced dermatologists, due to the wide range of morphologies in skin lesions. Given the rapid development of deep learning algorithms for melanoma diagnosis, it is crucial to validate and benchmark these models, which is the main challenge of this work. This research presents a new benchmarking and selection approach based on the multi-criteria analysis method (MCDM), which integrates entropy and the preference ranking organization method for enrichment of evaluations (PROMETHEE) methods. The experimental study is carried out in four phases. Firstly, 19 convolution neural networks (CNNs) are trained and evaluated on a public dataset of 991 dermoscopic images. Secondly, to obtain the decision matrix, 10 criteria, including accuracy, classification error, precision, sensitivity, specificity, F1-score, false-positive rate, false-negative rate, Matthews correlation coefficient (MCC), and the number of parameters are established. Third, entropy and PROMETHEE methods are integrated to determine the weights of criteria and rank the models. Fourth, the proposed benchmarking framework is validated using the VIKOR method. The obtained results reveal that the ResNet101 model is selected as the optimal diagnosis model for melanoma in our case study data. Thus, the presented benchmarking framework is proven to be useful at exposing the optimal melanoma diagnosis model targeting to ease the selection process of the proper convolutional neural network architecture.

## 1. Introduction

Skin cancer is the most frequent type of cancer and can be highly truculent [1]. In the UK, more than 100,000 new cases of skin cancer are reported each year [2]. In 2016, 1319 death cases from non-melanoma skin cancer and 2285 death cases from melanoma skin cancer were reported [3,4]. The initial stage in melanoma diagnosing is usually a visual assessment of the skin lesions. In comparison to inspection with the naked eye, dermatoscopy is one of the dermatologists’ most popular imaging procedures, and a frequently used diagnostic tool that enhances and improves the diagnosis of malignant and benign pigmented skin lesions [5].

A dermoscopy magnifies the surface of the skin lesion, allowing better visualization of deeper skin structures. It provides improved diagnostic accuracy of skin lesions, enabling the dermatologist to examine them more thoroughly. There are two main dermoscopy modes: non-polarized dermoscopy (NPD) and polarized dermoscopy (PD). Non-polarized dermoscopy (NPD) is integrated with a magnification lens and light-emitting diodes to provide illumination, enabling the visualization of subsurface structures in the epidermis. Non-polarized dermoscopy (NPD) requires direct contact of the glass plate with the skin surface and the presence of a liquid interface, such as alcohol, liquid paraffin, water, or ultrasound gel. The interface fluid dramatically increases the penetration of light, reduces scattered radiation, and produces a clear, low-reflection image, which allows excellent visualization of the superficial layers of the skin from the epidermis to the dermal–epidermal junction (DEJ). Like NPD, polarized dermoscopy (PD) contains light-emitting diodes to provide illumination and are equipped with a magnification lens. However, PDs use two polarized filters to achieve cross-polarization. NPD does not require direct contact with the skin and does not require the use of immersion liquids. PD allows visualization of subsurface structures located at the dermal–epidermal junction (DEJ) or superficial dermis. PD nearly blinds to the skin’s surface and structures in the superficial epidermis. Hence, non-polarized dermoscopy reveals superficial features, while polarized dermoscopy shows deeper structures, inferring that the use of both methods can provide complementary information [6,7]. Melanoma is diagnosed in two ways: visual inspection and biopsy. ABCDE (asymmetric, shape, border, color, diameter, and evolution) [8] are the main criteria used for visual screening of melanoma lesions based on a geometric description. Because the ABCDE approach is entirely dependent on the practitioner’s visual acuity and experience, this approach can be performed efficiently only by trained dermatologists [9].

However, manual review by dermatologists is a time-consuming, controversial, and error-prone task. The number of required dermatologists comparing the size of the population in the United States, Australia, and the UK is considerably low [10,11,12]. In the USA, the required number of dermatologists should be more than 4 per 100,000 individuals, which is the number that is suggested to adequately care for a population. However, it is currently estimated at 3.4 per 100,000 individuals. Similarly, there are just 550 practicing dermatologists in Australia, which is almost 15 % less than what is required to meet the needs of the population [11]. In the UK, the Royal College of Physicians (RCP) [13] recommends one full-time equivalent (FTE) consultant per 62,500 of the population. The RCP recommends 989 FTE consultant dermatologists. The British Association of Dermatologists (BAD) [14] found that there are 813 dermatology specialists in the UK Compared to the RCP’s recommendations, the BAD show a shortfall in the region of 250 consultants [12]. Hence, melanoma patients may not be aware of the severity of their disease if they do not undergo inspection by skilled specialists during an early stage of the disease and, thus, miss the ideal time to treat their conditions.

These obstacles encourage and inspire researchers to create automated melanoma diagnosis methods, using computer-aided diagnosis (CAD) systems. For non-experienced dermatologists, the CAD tool could provide a user-friendly environment, used as a second opinion in melanoma cancer diagnosis [15,16]. A large volume of skin images were collected in recent years, and sophisticated deep learning-based models [17] were successfully trained to perform automatic analysis of these skin images due to the industrial advancement of both computer hardware represented by graphics card capabilities and software technologies. These breakthroughs prompted expectations that automated diagnostic tools which will be available in the near future to examine and diagnose all types of skin lesions without the requirement of human experience [18].

Many automated melanoma diagnosis systems based on deep learning techniques, especially deep convolutional neural networks (CNNs), were recently developed. The new methods have significantly advanced the state of the art in skin lesion analysis. The CNN can automatically extract and learn high-level features, increasing the robustness of melanoma images’ inter- and intra-class variability [19,20]. With the rapid increase in the number of automatic recognition of melanoma from dermoscopy images using CNNs, comparing results among pieces of works and evaluation has become an awkward task. This limitation is due to methodological constraints and the absence of some of the standard metrics used to evaluate the performance of the models in terms of sensitivity, specificity, specificity, etc. To overcome these limitations, we assess and benchmark the deep learning models applied for melanoma diagnosis by considering similar methodological constraints, similar experimental settings and parameter setups, and similar evaluation criteria for all the deep learning models used in this study. Due to the existence of trade-offs and conflict among performance evaluation criteria during the evaluation process, the benchmarking of DL models is dealt with as a multiple criteria problem [21]. Accordingly, multi-criteria decision-making schemes (MCDM) can be exploited to benchmark the convolutional neural network models used for melanoma diagnosis.

Multi-criteria decision-making methods (MCDM) are an application of decision theory that handles multi-objective choice. It is a strategy for assessing and comparing multiple solutions (alternatives) considering competing criteria. It is a widely used decision-making approach in the field of operational research that deals with several criteria to find an optimal solution for decision makers. MCDM techniques find the optimal selection by ranking the performance of the alternatives, where the highest rank is assigned the best feasible alternative (solution) [21,22,23]. Two key problems could arise during the evaluation and benchmarking of deep convolutional neural network models for melanoma detection. First, what are the suitable criteria for the evaluation? Second, what is the proper benchmarking approach for selecting the optimal model considering the provided criteria?. Thus, the motivation of this work is to present a framework for evaluating and benchmarking multiple deep learning models for melanoma detection, using various evaluation criteria.

In light of the concerns mentioned above and given the rapid development of deep learning algorithms for melanoma diagnosis, it is crucial to validate and benchmark these models, which is the main challenge of this work. This research direction aims to conduct a comprehensive evaluation and benchmark of convolutional neural networks for melanoma diagnosis. The benchmarking is accomplished by prioritizing convolutional network architectures and then selecting the optimal architecture, given specific criteria. The contribution of our work can be represented in four-fold as follows:

- The proposed study provides an appropriate and powerful linkage between the multi-criteria decision-making techniques and the objective performance evaluation criteria, which are typically used to evaluate the deep learning models. This integration with decision-making schemes helps to rank the learning models based on multiple conflicting criteria and select the optimal model in our case study.
- This is the first study that introduces the application of a multi-criteria decision-making approach based on merging entropy and PROMETHEE methods to help prioritize the deep convolutional neural networks used for melanoma diagnosis and select the optimal model considering various criteria.
- This study presents a comprehensive evaluation of 19 convolutional neural network models with a two-class classifier. The models are trained and evaluated on a dataset of 991 dermoscopic images considering 10 performance evaluation metrics.
- The findings of our investigations would aid and expedite the timely deployment of artificial intelligence (AI)–assisted CAD systems to clinics and hospitals with regard to easing model selection under different criteria.

The remainder of this paper is presented as follows: in Section 2, the materials and proposed methods are described and explained; the experiments and results of the proposed system are designed, reported and discussed in Section 3; and finally, the work is concluded in Section 4.

## 2. Materials and Methods

### 2.1. Materials

**Figure 1:**
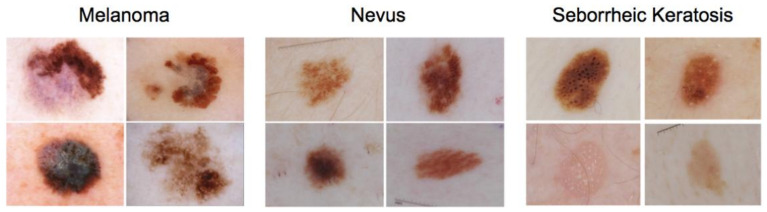
Example of images used to conduct this study. Both nevus and seborrhoeic keratosis are classified as non-melanoma in our experiments.

To carry out our experiments, dermoscopic images were collected from the openly available International Skin Imaging Collaboration (ISIC 2017) dataset [18]. Melanoma, seborrheic keratosis, and nevus, shown in Figure 1, are the three types of lesions represented in the dataset. Melanoma is a cancerous skin tumor with a high mortality rate. Seborrheic keratosis and nevus, the other two types of lesions, are benign skin tumors formed from different cells. Although the ISIC Challenge 2017 included three subtasks with annotations for three classes (nevus, seborrheic keratosis, and melanoma), we only consider the melanoma subtask versus the remaining classes, producing a two-class classification task. The ISIC (2017) dataset comprises 2000 training images and 600 test images. In the training set, there are 374 melanoma images and 1626 non-melanoma images. The test set contains 117 melanoma images and 483 non-melanoma images. In total, both training and test data comprise 491 melanoma images and 2109 non-melanoma images. The percentage of melanoma images in the dataset is 19%. This ratio shows a highly imbalanced data distribution between the two classes. Our study does not target to develop a new method for melanoma diagnosis competing with other methods in which particular strategies are designed to remedy and alleviate the effect of imbalanced data. Instead, this study aims to evaluate and benchmark the existing CNNs architectures considering multiple conflicting criteria. The condition of benchmarking in this study is set for balanced data. Thus, to maintain the balance of classes distribution, all the melanoma images (491) in the dataset are collected, whereas only the first 500 non-melanoma images are gathered, producing 991 dermoscopic images in total. The data are split into five folds for training and testing. In each of the five training cycles, four folds are used for training, and the hold-out set is used for testing the network performance. Thus, in each training process, this generates 393 images (melanoma) and 400 images (non-melanoma) for training, and 98 images (melanoma) and 100 images (non-melanoma) for testing.

### 2.2. Methods

**Figure 2:**
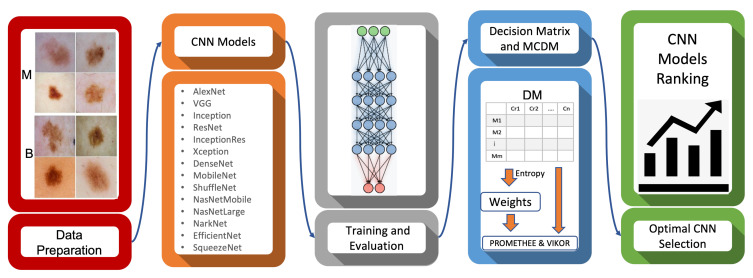
The block diagram of the proposed framework used to benchmark CNN models for melanoma diagnosis. M refers to malignant (melanoma) and B refers to benign (non-melanoma).

Our developed evaluation and the benchmarking system illustrated in Figure 2 comprises five main stages, including data preparation, designing of CNN models, training of CNN models, evaluation criteria establishment, and benchmarking of CNN models using MCDM. In the first and second phases of the proposed framework, depicted as red and orange blocks in Figure 2, the data are prepared, and deep convolutional neural networks are implemented (different versions of a specific CNN architecture are considered; for instance; VGG16, VGg19). In the third phase, depicted as a grey block, the CNN models are trained. The key evaluation criteria are identified and measured by evaluating the trained models on test data. In the final phases, shown as blue and green blocks, MCDM methods are employed to prioritize the alternatives (i.e., CNN models). The blue block shows the construction of the decision matrix (models as rows and criteria as columns); then, the entropy method is applied to calculate and generate the weights of criteria. Finally, the MCDM methods (PROMETHEE and VIKOR) are exploited to rank CNN models and report the optimal CNN architecture considering the provided decision matrix and the weights of criteria. Although PROMETHEE and VIKOR are different statistical methods, the input data of these methods are the same, which are the weights of criteria and the decision matrix. These methods are independent; therefore, they are applied to the given input data separately. In this section, each phase of the proposed framework is described as follows:

#### 2.2.1. Pre-Trained Convolutional Neural Network Models (CNNs)

The key CNN baseline architectures that have been applied in this study are summarized below:

- AlexNet: In 2012, AlexNet [24] substantially surpassed all previous classification methods, winning the ImageNet Large Scale Visual Recognition Competition (ILSVRC) by reducing top-5 error from 26% to 15.33%. The network’s design was similar to the LeNet network developed by Yann LeCun et al. [25], but it was deeper, with more filters per layer and layered convolutional layers. 11×11, 5×5, 3×3 convolutions filters, max pooling, dropout, data augmentation, ReLU activations, and SGD with momentum were all included. After each convolutional layer, added ReLU activations were added. AlexNet was trained using two Nvidia Geforce GTX 580 GPUs for six days, which is why their network is divided into two pipelines.
- VGG16,19: Simonyan and Zisserman presented the VGG architecture in 2014 [26]. It is a straightforward design, with only blocks made up of an incremental number of convolution layers and 3 × 3 filters. Furthermore, max-pooling blocks follow convolution blocks to reduce the size of the activation maps obtained. Finally, a classification block is employed, consisting of two dense layers and a final output layer. The numbers 16 and 19 refer to how many weighted layers each network includes. On the other hand, this network has a couple of drawbacks: it takes too long to learn and has a lot of parameters.
- InceptionV1,V3: Google implemented inception building blocks in GoogLeNet (Inceptionv1) [27]. These blocks function well together and result in a model that is easy to generalize. GoogLeNet is made up of nine Inception modules that are stacked one on top of the other. There are a total of 27 layers, 5 of which are pooling layers. The total number of layers used in the network design is about 100. New revisions of the model appeared as the model was updated regularly. Inception-v2 and Inception-v3 [28] were released within a short time gap in 2015. Except for a few features, Inception-v2 integrates all of GoogLeNet’s features. Filter banks were increased in width in Inception-v2 to eliminate the “representational bottleneck”. All of the changes from Inception-v2 were included in Inception-v3. Furthermore, Inception-v3 underwent additional changes, such as the use of a higher resolution input and the use of the RMSProp optimiser, which significantly reduced the cost function.
- InceptionResNetV2: Inception V4 was launched in 2016 by Google researchers in conjunction with Inception-ResNet. By implementing Inception-V4, the main goal of this network architecture was to reduce the complexity of the Inception V3 model, which provided state-of-the-art accuracy on the ILSVRC2015 challenge. This architecture also investigates the use of residual networks on the Inception model [29].
- ResNet18,50,101: The ResNet architecture, founded by He et al. in 2015 [30], was a major turning point in the introduction of an extraordinary form of architecture focused on “modules” or “networks within networks”. The principle of residual connections was first implemented in these networks. ResNet comes in various sizes and numbers of layers—such as ResNet18, RerNet50, and RerNet101—but the most common is ResNet50, which has 50 layers with weights. Despite having many more layers than the VGG, ResNet50 needs nearly five times less memory. This is because, instead of dense layers, this network uses a layer called GlobalAveragePooling in the classification stage, which transforms the 2D feature maps of the last layer in the feature extraction stage into an n-classes vector that is used to measure the likelihood of belonging to each class.
- DenseNet201: DenseNet [31] is very similar to ResNet, but there are a few key differences. DenseNet concatenates the output of the previous layer with the output of the next layer. At the same time, ResNet follows an additive approach that combines the previous layer (identity) with the next layer. DenseNet model was founded mainly to address the vanishing gradient’s impact on high-level neural networks’ layers. Using the composite function operation, the previous layer’s output becomes the second layer’s input. Convolution, pooling, batch normalization, and non-linear activation layers form this composite process. DenseNet comes in a variety of types, including DenseNet-121, DenseNet-169, and DenseNet-201. The numbers represent the number of the neural network’s layer.
- Xception: Xception [32] is an extension of the Inception architecture that uses depthwise separable convolutions to replace the regular Inception modules. The mapping of cross-channel and spatial correlations in the feature maps of convolutional neural networks can be fully decoupled in this network. The authors called their proposed architecture Xception, which stands for “Extreme Inception,” since this hypothesis is a stronger version of the hypothesis that underlies the Inception architecture. In a nutshell, the Xception architecture is a depthwise separable convolution layers stack with residual connections. This makes it very simple to establish and change the architecture.
- MobileNet: MobileNet [33] is a convolutional neural network designed for mobile and embedded vision uses. They are based on a streamlined architecture that builds lightweight deep neural networks with low latency for mobile and embedded devices, using depthwise separable convolutions. The width multiplier and resolution multiplier parameters are added to make it easier to tune MobileNet. The depthwise convolution in MobileNets applies a single filter to each input channel. After that, the pointwise convolution applies a 1×1 convolution to combine the depthwise convolution’s outputs. A separate layer for filtering and a separate layer for combining are used in depthwise separable convolution. This factorization has the effect of reducing the computation and model size drastically.
- NASNetMobile and NASNetLarge: Google Brain built Neural Architecture Search (NASNet) [34]. The authors suggested that an architectural building block be detected on a small dataset and then transferred to a larger dataset. They generally look for the best convolutional layer or cell on a small dataset first, then stack together more copies of this cell to extend to the larger dataset. A new regularization technique called ScheduledDropPath was proposed, which significantly enhances the generalization of the NASNet models. With a smaller model size and lower complexity, the NASNet method achieves state-of-the-art results. While the overall architecture of NASNet is predefined, the blocks or cells are not. Alternatively, a reinforcement learning search technique is used to find them. The authors developed different versions of NASNets with different computational requirements. The larger model, NASNetlarge, is a convolutional neural network trained on over onen million images from the ImageNet database, while the smaller model, NASNetMobile, is optimized for mobile devices.
- ShuffleNet: ShuffleNet [35] is a convolutional neural network optimized for mobile devices with minimal processing capacity developed by Megvii Inc. (Face++). The network architecture design considers two new operations to lower computation costs while retaining accuracy: pointwise group convolution and channel shuffle. It specializes in common mobile platforms, such as drones, robots, and smartphones, and aims for the best accuracy in minimal computational resources.
- DarkNet19,53: The backbone of YOLOv2 is a convolutional neural network called Darknet-19 [36]. It generally employs 3×3 filters and twice the number of channels after each pooling phase, similar to VGG models. It leverages global average pooling to produce predictions and 1×1 filters to compress the feature representation among 3×3 convolutions, identical to the work on Network in Network (NIN). Batch normalization is a technique for stabilizing training and accelerating convergence. Darknet-53 [37], on the other hand, is a convolutional neural network that serves as the backbone for the YOLOv3 object detection method. The utilization of residual connections and more layers are an enhancement over its predecessor, Darknet-19.
- EfficientNetB0: EfficientNetB0 [38] is a convolutional neural network that scales depth, width, and resolution dimensions, using a compound coefficient. Unlike the traditional methodology, which arbitrarily scales network dimensions, the EfficientNetB0 scaling strategy scales network dimensions with a set of predetermined scaling coefficients. According to the compound scaling approach, if the input image is larger, the network needs more layers and channels to widen the receptive field and catch more fine-grained patterns on the larger image. In addition to squeeze-and-excitation blocks [39], the base of EfficientNet is built on MobileNetV2’s inverted bottleneck residual blocks [33].
- SqueezeNet: DeepScale, UC Berkeley, and Stanford University collaborated to develop SqueezeNet [40]. With 50× fewer parameters, SqueezeNet reaches AlexNet-level accuracy on ImageNet. Additionally, the authors were able to compress SqueezeNet to less than 0.5 MB, using model compression approaches (510× smaller than AlexNet). Smaller convolutional neural networks (CNNs) require less communication across servers during distributed training and less bandwidth. They are also more feasible to be deployed on FPGAs and hardware with restricted computational resources and limited memory.

#### 2.2.2. Benchmarking Criteria

This section presents elaboration for the criteria taken into consideration in this study. The choice of criteria in MCDM methods is highly dependent on the decision-making context, and the problem handled. As we deal with a classification problem, our study has established the most popular measurements typically used for classifiers’ evaluation as criteria. The performance of each CNN model was evaluated in this stage, using 10 evaluation metrics. We utilized the test accuracy, F1-score, sensitivity, specificity, precision, false-positive rate and false-negative rate, Matthews correlation coefficient (MCC), classification error, network complexity to evaluate each of the model targeted for study in this research.

- Accuracy: this metric measures how close the predicted value is to the actual data values. It can be defined using the following formula:
  (1) $$Accuracy\;\left(Acc\right)=\frac{tp+tn}{tp+tn+fp+fn}$$
  tp: True Positive, tn: True Negative, fp: False Positive, fn: False Negative
- Classification error: This refers to the number of samples incorrectly classified (false positives and false negatives). It can be defined as follows:
  (2) ClassificationError(Err)=1−Acc
- Precision: The precision metric tests the ability of the classifier to reject irrelevant samples. The formula of this metric can be defined as follows:
  (3) $$Precision\;\left(Pre\right)=\frac{tp}{tp+fp}$$
- Sensitivity: The sensitivity metric measures the proportion of the correctly detected relevant samples. It can be represented as follows:
  (4) $$Sensitivity\;\left(Sn\right)=\frac{tp}{tp+fn}$$
- F1-Score: The F1-score can be obtained by the weighted average of sensitivity (recall) and precision, where the relative contribution of both recall and precision to the F1-score are equal. The F1-score can be defined as follows:
  (5) $$F_{1}\;Score=\frac{2(Precision\times Recall)}{Precision+Recall}$$
  where Recall = Sensitivity
- Specificity: It describes the ability of the classifier to detect the true negative rate. The formula of specificity can be defined using the following equation:
  (6) $$Specificity\;\left(Sp\right)=\frac{tn}{tn+fp}$$
- False-Positive Rate (FPR): This is the proportion of negative examples wrongly categorized as positive. This metric is also known as the miss rate and is represented as follows:
  (7) $$False-Positive\;Rate\;\left(FPR\right)=\frac{fp}{fp+tn}$$
- False-Negative rate (FNR): This is the proportion of negative examples wrongly categorized as positive. This metric is also known as the fall-out rate. This evaluation criterion is introduced as follows:
  (8) $$False-Negative\;Rate\;\left(FNR\right)=\frac{fn}{fn+tp}$$
- Matthews Correlation Coefficient (MCC): The MCC is a correlation coefficient that yields a value between −1 and +1 for actual and estimated binary classifications. A coefficient of +1 shows ideal prediction, 0 shows random prediction, and −1 indicates complete disagreement between predictions and the ground truth. The MCC can be defined as follows:
  (9) $$MCC=\frac{\left(t_{p}\times t_{n}-f_{p}\times f_{n}\right)}{\sqrt{\left(t_{p}+f_{p}\right)\left(t_{p}+f_{n}\right)\left(t_{n}+f_{p}\right)\left(t_{n}+f_{n}\right)}}$$
- CNN Complexity: This refers to the number of parameters existing in the pre-trained CNN.

#### 2.2.3. Multi-Criteria Decision Making (MCDM)

Multi-criteria decision making typically involves six phases: (i) problem formulation, (ii) identification of requirements, (iii) goal setting, (iv) identification of alternatives, (v) development of criteria, and (vi) the identification and application of decision-making techniques. This process can be carried out using various mathematical procedures chosen based on the problem at hand, and the level of complexity ascribed to the decision-making process [41,42]. This study has formulated the CNN models benchmarking as the research goal, considering 19 CNNs as alternatives and 10 criteria. For decision making, preference ranking organization method for enrichment evaluation (PROMETHEE) [43], an MCDM method, is adopted to generate the ranking list and to produce the optimal model selection, using the criteria’s weights computed by the entropy method. For validating the optimal model selection, another MCDM method called VlseKriterijumska Optimizacija I Kompromisno Resenje (VIKOR) in Serbian [44], which means multi-criteria optimization and compromise solution, is also applied. This section describes the MCDM methods exploited to rank the CNN models and selects the optimal model, given the criteria mentioned earlier, using the data in our case study.

- Entropy: This method computes relative weights by objectively interpreting the relative intensities of the criteria significance based on data discrimination [45]. MDCM’s generated decision matrix DM is defined by *m* alternatives (19 CNN models) and *k* criteria (10 criteria), which are represented as follows:
  (10) $$DM={\left[x_{ij}\right]}_{m\times k}$$
  From the constructed decision matrix DM, the procedure of entropy weighting method described in [45] is followed to measure the weights $w_{j}$. $x_{ij}$ refers to each entry in the DM, where i=1,…,m, j=1,…,k. The steps of the entropy weighting method [45] are described as follows:
  **Step1:** Normalizing the decision matrix using the following equation:
  (11) $$p_{ij}=\frac{x_{ij}}{\sum_{i=1}^{m}x_{ij}},\left(1\leq i\leq m,1\leq j\leq k\right)$$
  **Step2:** Measuring the entropy value for each criterion as follows:
  (12) $$\begin{array}{c} e_{j}=-g\sum_{j=1}^{k}p_{ij}\ln p_{ij},\left(g=1/\ln m,1\leq i\leq m,1\leq j\leq k\right). \end{array}$$
  **Step3:** Determining the inherent contrast intensity of each criterion as follows:
  (13) $$d_{i}=1-e_{j},\left(1\leq j\leq k\right)$$
  **Step4:** The entropy weights of criteria are then defined as follows:
  (14) $$w_{j}=d_{j}/\sum_{j=1}^{k}d_{j},\left(1\leq j\leq k\right)$$
- PROMETHEE: The PROMETHEE is an outranking approach for ranking and selecting a finite collection of alternatives based on often competing criteria. Compared to other multi-criteria analysis methods, PROMETHEE II is an uncomplicated complete (not partial) ranking method in terms of conception and application. The stepwise procedure of PROMETHEE II can be defined as follows, giving the provided decision matrix and the weights of criteria:
  **Step 1:** Determining of deviations based on pairwise comparisons as follows:
  (15) $$d_{j}\left(a,b\right)=g_{j}\left(a\right)-g_{j}\left(b\right)$$
  where $d_{j}\left(a,b\right)$ refers to the difference between the evaluations of *a* and *b* on each criterion.
  **Step 2:** Preference function application:
  (16) $$P_{j}\left(a,b\right)=F_{j}\left[d_{j}\left(a,b\right)\right]\;j=1,\ldots ,k$$
  where $P_{j}\left(a,b\right)$ denotes the preference of alternative *a* with regard to alternative *b* on each criterion, as a function of $d_{j}\left(a,b\right)$.
  **Step 3:** Calculating an overall or global preference index using the following formula:
  (17) $$\pi \left(a,b\right)=\sum_{j=1}^{k}P_{j}\left(a,b\right)w_{j}$$
  where π(a,b) of *a* over *b* represents the weighted sum p(a,b) for each criterion, and $w_{j}$ is the weight $w_{j}$ related to the *j* th criterion.
  **Step 4:** Calculating the partial ranking PROMETHEE I (outranking flows) using the following equations:
  (18) $$\phi^{+}\left(a\right)=\frac{1}{m-1}\sum_{b=1}^{m}\pi \left(a,b\right)$$
  (19) $$\phi^{-}\left(a\right)=\frac{1}{m-1}\sum_{b=1}^{m}\pi \left(b,a\right)$$
  where $\phi^{+}\left(a\right)$ and $\phi^{-}\left(a\right)$ represent the positive outranking flow and negative outranking flow for each alternative, respectively.
  **Step 5:** Calculating the complete ranking PROMETHEE II (outranking flows) using the following equations:
  (20) $$\phi \left(a\right)=\phi^{+}\left(a\right)-\phi^{-}\left(a\right)$$
  where ϕ(a) represents the outranking flow for each alternative.
- VIKOR: The VIKOR approach [44] was initially developed to optimize complex systems that involve various parameters. Using the predefined weights, the VIKOR provides a compromise ranking list and suggests a compromise solution. VIKOR creates a multi-criteria rating index based on a specific “closeness” metric to the “ideal” solutions [44]. The VIKOR methodology’s compromise ranking algorithm can be described as follows, giving the provided decision matrix and the weights of criteria.
  **Step1:** Determining the best value as ${x_{j}}^{*}$ and the worst value as ${x_{j}}^{-}$ of the criteria as j=1,2,⋯,k. This also leads to configure the criteria as beneficial and non-beneficial values. The beneficial attributes require being maximized, while the non-beneficial ones need to be minimized, which are identified as follows:
  **Rule1:** Best value for beneficial criteria is ${x_{j}}^{*}=maxx_{ij}$, and for non-beneficial is ${x_{j}}^{*}=minx_{ij}$,
  **Rule2:** Worst value for beneficial criteria is ${x_{j}}^{-}=minx_{ij}$, and for non-beneficial is ${x_{j}}^{-}=maxx_{ij}$.
  **Step2:** Determining the values of $S_{i}$ and $R_{i}$, where i=1,2,⋯,m using the following equations:
  (21) $$\begin{array}{c} S_{i}=\sum_{j=1}^{k}w_{j}\left(x_{j}^{*}-x_{ij}\right)/\left(x_{j}^{*}-x_{j}^{-}\right),\\ R_{i}=\max_{j}w_{j}\left(x_{j}^{*}-x_{ij}\right)/\left(x_{j}^{*}-x_{j}^{-}\right), \end{array}$$
  where $w_{j}$ are the weights of criteria computed using the entropy method.
  **Step3:** Determining the values of $S^{*}$ and $R^{*}$ as follows:
  (22) $$\begin{array}{c} S^{*}=\min_{i}S_{i},R^{*}=\min_{i}R_{i},\\ S^{-}=\max_{i}S_{i},R^{-}=\max_{i}R_{i} \end{array}$$
  **Step4:** Determining the values of $Q_{i}$; where i=1,2,…,m and *v* is defined as the weight of the scheme of “the majority of criteria” using the following equation:
  (23) $$Q_{i}=v\left(S_{i}-S^{*}\right)/\left(S^{-}-S^{*}\right)+\left(1-v\right)\left(R_{i}-R^{*}\right)/\left(R^{-}-R^{*}\right)$$
  **Step5:** Ranking the alternatives by sorting the values of $Q_{i}$ in ascending order.

## 3. Experimental Results and Discussion

### 3.1. Experimental Setup and Training

**Figure A1:**
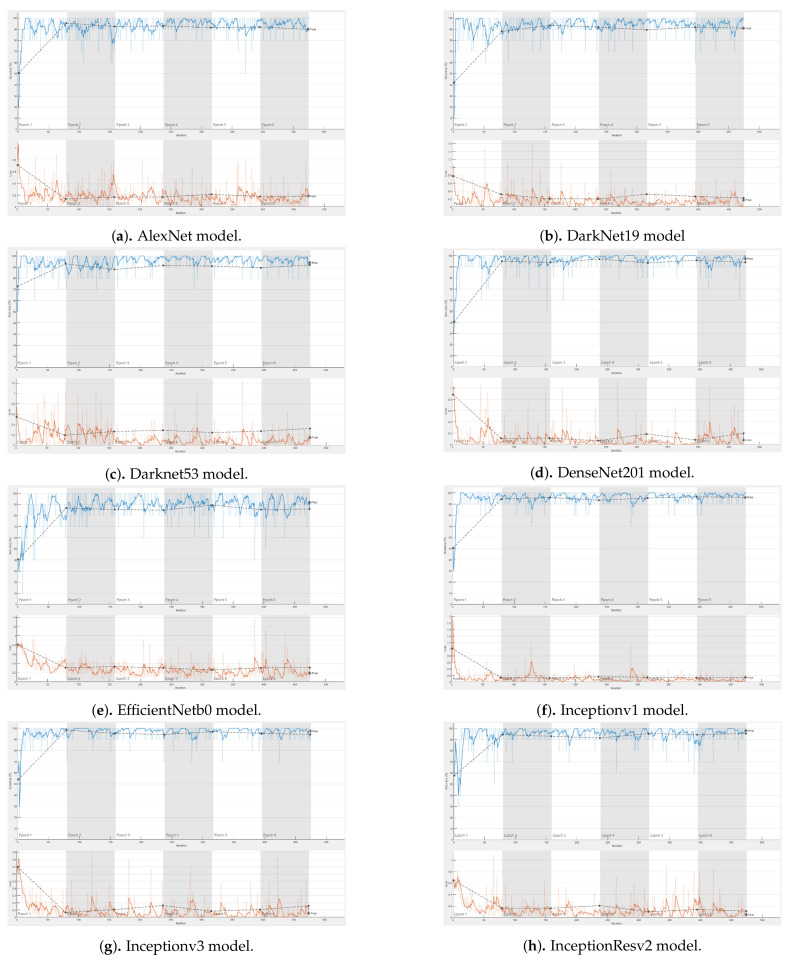

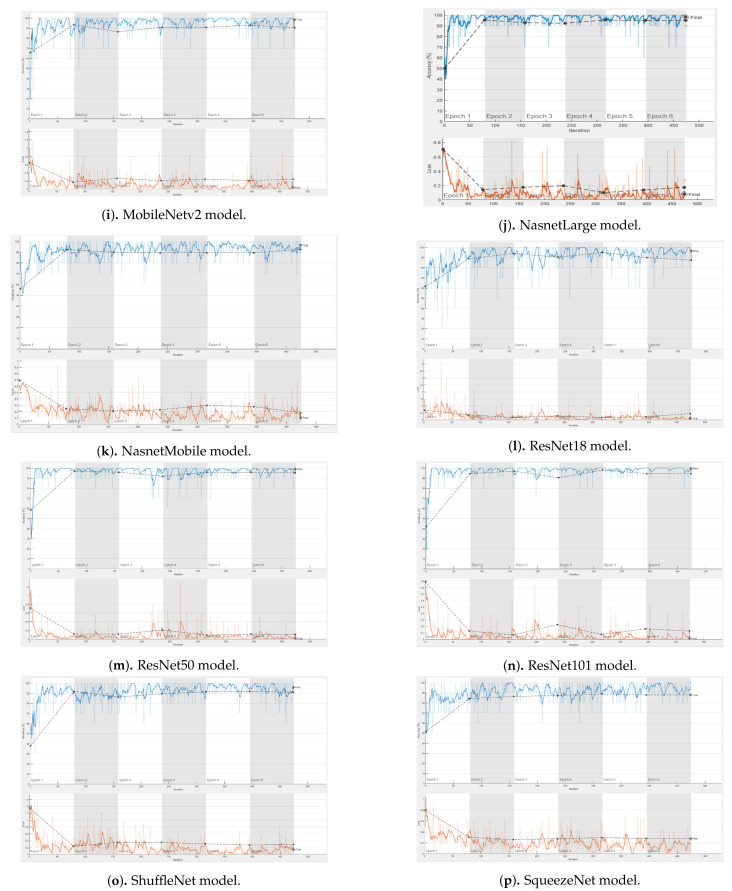

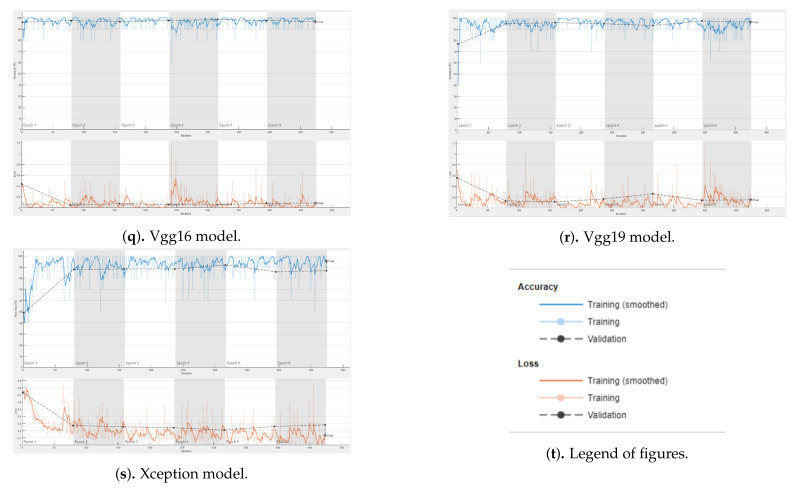
The performance of the CNN models visualizing training, validation and loss curves.

During the experimental process, 19 CNN models pre-trained on ImageNet dataset [46] were modified and re-trained using transfer learning and fine-tuning strategies to classify the skin lesion into two classes: cancerous (melanoma) or non-cancerous (non-melanoma). The characteristics of the CNN architectures in terms of number of total layers, number of learnable layers, size of CNN, size of the input image, and number of parameters in each network architecture are described in Table 1. In the training of models, binary cross-entropy was preferred as a cost function, and the stochastic gradient descent with momentum (SGDM) optimizer to minimize the cost function. The softmax activation function was used in the output layer of the models. Each model was trained through six epochs, and the training was repeated for a total of five times. The batch size is set to 10, providing 79 iterations per epoch and 474 iterations for six epochs. The learning rate value was set to 0.0003 and momentum of 0.9. The learning curves of 19 CNN models are presented in Figure A1.

To provide fair performance evaluation and benchmarking among the nineteen models, we opted to use a fixed number of epochs for all models. Figure A1 shows that all the models stopped training at the same endpoint, and the trained models were deployed from this endpoint to conduct the testing phase. We aimed to compare the performance of the networks under the same constraints and conditions. So, choosing the optimal number of epochs to train a particular model was not considered. Considering learning the models under the same conditions, if one model encounters overfitting and subsequently fails to achieve good accuracy on the unseen test set, whereas another model has not undergone overfitting, the later model is preferred over the former model. However, in Figure A1, it can be noticed that the training and validation curves show a steady learning behavior, and there is no indication of overfitting. In order to prevent potential overfitting during the training, the online data augmentation is applied by using various image transformation methods, such as vertical and horizontal flipping, random translation in the range of [−30,30], and random scaling in the range of [0.9,1.1].

In most of the CNN models, the last layer is the learnable weights of fully connected layers. Thus, to apply the transfer learning and fine-tune the network, using our data, these completely connected layers are replaced with a new, fully-connected layer, comprising two neurons adhering to the two classes in our study. Instead of fully connected layers, the last learnable layer in some networks, such as SqueezeNet, is a 1×1 convolutional layer. In this scenario, the old convolutional layer is replaced by a new convolutional layer with the same number of filters as classes.

### 3.2. Results of the Experiments and Discussion

To examine the classification performance of the models, nine evaluation metrics widely used in classification tasks are used, including accuracy, classification error, precision, sensitivity, specificity, F1-score, false-positive rate, false-negative rate, and Matthews correlation coefficient. Table 2 depicts the evaluation performance of the 19 CNN models describing the average value and the standard deviation of a specific criterion over the five folds. This study reveals the high evaluation performance of the CNN models for melanoma diagnosis, employing a balanced number of dermoscopic images through a thorough analysis of 19 pre-trained CNNs using a specific parameter configuration and learning technique for the networks.

**Figure 3:**
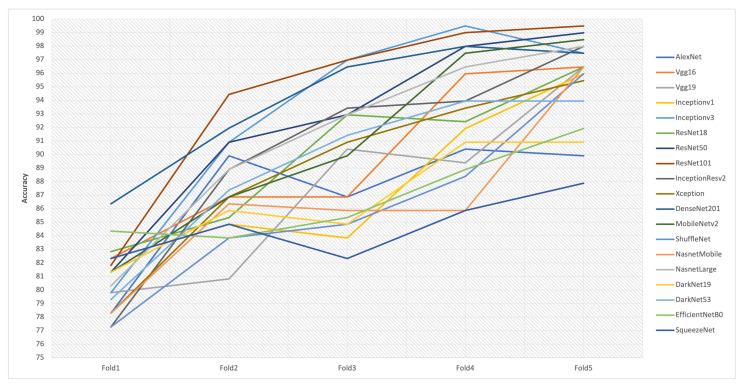
The obtained accuracies over five folds in the nineteen CNN models. It shows that there is no superior CNN model over others due to the lack of a CNN model that achieves the best accuracies through the five folds. This would lead to difficulty selecting the best model while considering another conflicting criterion, such as the network complexity.

As shown in Table 2, the ResNet101 model reported the best average test accuracy and MCC with 94.34% and 88.96%, respectively, compared to other CNN models. The highest F1-score with a value of 93.96% was attained by Densenet201, followed by ResNet101 with a value of 93.89%. Furthermore, Inceptionv3 achieved the highest specificity and precision values with 96.8% and 96.11%, followed by 96% specificity achieved by MobileNetv2 and 95.36% precision achieved by ResNet101. DenseNet201 produced the highest sensitivity of 93.47%, followed by 92.86% reported in ResNet101. It can also be noticed that Inceptionv3 attained the lowest FPR of 3.2%, while DenseNet201 revealed the lowest FNR of 6.53%, and the smallest error, 5.66%, was reported by ResNet101. According to the minimum number of parameters, SqueezeNet has 1.24 million parameters, which is the optimal number, compared to other CNN models. Table 2 also explores the deviation among the accuracies reported from the five folds and exposes the difficulty in recognizing the best model based on the variation of the accuracies in the five folds. Likely, Table 3 and Figure 3 show that there is no superior CNN model over others, due to the lack of a CNN model that achieves the best accuracies through the five folds. This would lead to difficulty in selecting the best model, while considering other criteria.

**Figure 4:**
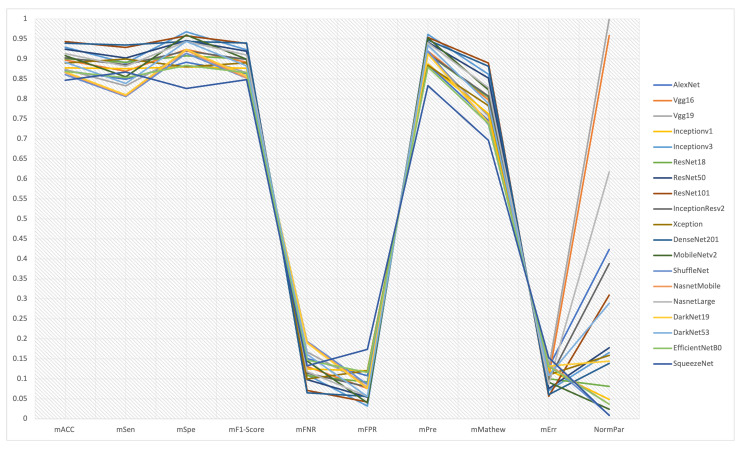
The mean value over the five folds for specific evaluation criteria, along with the number of parameters (the network complexity). No single model achieves the best performance in all evaluation criteria. If a CNN model achieves the best evaluation performance in some evaluation criteria, it may fail to gain superior performance in the remaining criteria.

Figure 4 exhibits the trade-off and conflict among the evaluation criteria of the 19 CNN models. For instance, a trade-off between sensitivity (true positive rate) and specificity (true negative rate) should be considered, where DenseNet201 reports the highest sensitivity, whereas Inceptionv3 attains the highest specificity. Precision is also independent and has a trade-off with accuracy. Accuracy is the degree of veracity, while precision is the degree of reproducibility. That means that it is possible to be very precise but not very accurate, and it is also possible to be accurate without being precise. The best quality detection is both accurate and precise. Inceptionv3 achieves the highest precision, whereas Resnet101 reveals the best accuracy. It should also produce a trade-off between FNR and FPR, where Inceptionv3 reports the lowest FPR, while DenseNet201 reports the lowest FNR. Thus, it is crucial to make a trade-off between the models that could achieve the optimal diagnosis by reducing the number of negative cases falsely diagnosed as positive and the models that could reach the optimal diagnosis by reducing the number of positive instances falsely diagnosed as negative. The F1-score is also needed to achieve a balance between precision and sensitivity, where Densenet201 provides the best F1-Score followed by Resnet101. For the number of parameters required to determine the network complexity, SqueezeNet has the lighter network architecture, compared to VGG19, which has the largest network architecture. Although SqueezeNet is optimal in terms of network complexity, it still shows moderate-low accuracy performance through the five folds shown in Figure 3. Additionally, there is a conflict between the criteria that are required to be minimized (such as FNR, FPR, Err, and the number of parameters) and the criteria targeted to be maximized (such as Acc, Sen, Spe, Pre, F1-score, and Mathew).

From Figure 4, it can also be noticed that there is no superior CNN model, due to the conflict among evaluation criteria and the difficulty to optimize all criteria simultaneously. Hence, selecting the best deep learning model for automated melanoma diagnosis considering multiple conflicted criteria is a difficult task, due to the variance of the criteria significance, the conflict among these criteria, and the trade-off among them. Therefore, benchmarking CNN architectures for melanoma detection is crucial for selecting the optimal model, achieving a trade-off among the 10 pre-defined evaluation criteria. The multiple criteria decision-making method (MCDM) [43,44] is targeted to apply and rank the 19 models according to their performance, considering the trade-off among the criteria. Thus, the best-selected networks could be easily adopted to construct an ensemble learning system for melanoma diagnosis or even use the optimal network to construct a system using a single model.

To achieve the goal of our study by generating a ranking list for CNN models and selecting the optimal solution, the PROMETHEE method [43] is applied considering the 19 alternatives (CNN models) and 10 criteria. To further validate the decision made by PROMETHEE, we also applied the VIKOR approach [44] using the same data setting and configuration. First, the decision matrix DM is constructed using *m* alternatives, in our case 19, and the *k* criteria, in our case 10, producing DM of size 19×10. The criteria are then classified into two categories according to the required optimization strategy. The first category includes the criteria that require minimization, including classification error, false-positive rate, false-negative rate and number of parameters, known as non-beneficial criteria. Unlikely, the second category includes the criteria that require maximization, including accuracy, sensitivity, specificity, precision, F1-score and MCC, known as beneficial criteria. The Equations (24) and (25) defined below are used for normalizing the non-beneficial and beneficial criteria, respectively. The normalized criteria are shown in Table 4.

(24) $${\bar{x}}_{ij}=\frac{x_{j}^{\min}}{x_{ij}}$$

(25) $${\bar{x}}_{ij}=\frac{x_{ij}}{x_{j}^{\max}}$$

$x_{ij}$ refers to the entries of the decision matrix DM, where i=1,…,m, j=1,…,k, *k* represents the number of alternatives (19 CNN models), and *m* defines the number of criteria (10 criteria).

To measure the weights of criteria, the entropy method [45] is exploited and applied on the normalized DM producing the weight values of 0.964825438, 0.804398756, 0.985470611, 0.951881312, −1.420375792, −1.473036988, 1.02152041, 0.49110277, −1.294287661, −0.031498856 for accuracy, sensitivity, specificity, F1-score, FNR, FPR, precision, MCC, classification error and number of parameters, respectively. The obtained weights, along with the normalized DM, are used to make the optimal selection, using the PROMETHEE method [43]. The equations used to measure the ranking list are described earlier in Section 2.2.3. We have used the threshold function as the preference function (0 if d ≤ 0 and 1 if d ≥ 0) required in Step 2 in the stepwise procedure of PROMETHEE. To calculate the complete ranking list, ϕ(a) represents the outranking flow for each alternative as shown in Table 5. The highest ϕ(a) value indicates the compromised solution, which could be chosen as the optimal model. PROMETHEE reports a value of 150.84, the highest ϕ(a) for the ResNet101 CNN model and 133.24 as the second-best value for the DenseNet201 model.

To validate the model selection made by PROMETHEE, the VIKOR [44] method is also applied, considering the same weights and the same DM. Unlike PROMETHEE, the lowest *Q* value in VIKOR indicates the compromised solution, which could be chosen as the optimal model, shown in Table 5. VIKOR reports a value of 0, the lowest *Q* for the ResNet101 CNN model, and 0.079 as the second-lowest value for the DenseNet201 model. Thus, the mathematical consistency of the judgements coming out of PROMETHEE II was tested and proven. Hence, the effectiveness of the model ranking produced by PROMETHEE II was validated by demonstrating the agreement between two different statistical methods, considering the same conflicting criteria.

To provide a direct and explicit comparison between the two decision-making methods, PROMETHEE and VIKOR, Table 6 elaborates the optimal CNN model selection in both approaches. It can be noticed that until the seventh rank, the two methods have a similar decision for the optimal CNN model selection. Likewise, the ranks 10, 11, 12, 13, 15, 18 and 19 provide the exact model recommendation by both approaches. On the other hand, the decision made by methods has slightly different priorities for the 8, 9, 14, 16 and 17 levels. The suggested framework’s findings show that the best model selection decision based on numerous conflict factors is robust and reliable.

This work developed a new multi-criteria decision-making methodology that aids in assessing the criteria that influence the decision to choose a specific CNN model, prioritizing the models and selecting the best model. When software developers need to find an effective CNN model that meets specified requirements for constructing a robust CAD system, the proposed approach of revealing the CNN models’ priorities would be beneficial and valuable. Finally, our study may provide and draw a new line in the evaluation and benchmark of the deep learning models for various diseases. Although the proposed benchmarking framework has made progress in benchmarking the models used for melanoma diagnosis from dermoscopy images, there is still space for improvement in research work. In future work, we aim to study the effect of the model selection, considering different criteria. The criteria that are to be considered include (i) training the models under several transfer learning scenarios and data augmentation strategies, (ii) exploring the impact of several optimization schemes, and (iii) testing various class balancing and weighting techniques. We also consider training the models on several datasets, targeting the effect of variation among datasets. These reported limitations and suggested improvements are currently part of the authors’ ongoing research.

## 4. Conclusions

Medical diagnostics tools based on deep learning of medical images are becoming more widely recognized as clinically relevant AI-based solutions. However, developing appropriate deep neural network models and training strategies for clinical uses is a research area that needs to be investigated. The inaccurate selection of melanoma diagnosis model could be costly to medical organizations, especially when more accurate and efficient diagnosis models are urgently needed. This study investigated the performance of some of these networks for melanoma diagnosis, utilizing dermoscopic images after a thorough evaluation of 19 pre-trained CNNs, using particular evaluation criteria, parameter settings and training strategies. An MCDM-based methodology is presented for evaluating, benchmarking, and ranking melanoma diagnostic models and selecting the most optimal model. The study findings would help in the model selection, designing quick and reliable diagnostic tools based on image data, and contributing to the development of more accurate and efficient point-of-care diagnostic and detection systems. Other image modalities, such as non-dermoscopic (clinical) images, can also be used to train and test the network architecture of the pre-trained models. Therefore, we aim to adapt our proposed network designs in the future to include not only dermoscopic but also clinical images. We would also like to expand the number of training samples and investigate other deep learning training methodologies.

## Figures and Tables

**Table 1:** Characteristics of the pre-trained CNN architectures adopted in our study.

Network	#Layers	#Learnable Layers	Network Size (MB)	Input Image Size	#Para (Millions)
AlexNet [24]	25	8	227	227×227	61
Vgg16 [26]	41	16	515	224×224	138
Vgg19 [26]	47	19	535	224×224	144
GoogleNet (Inceptionv1) [27]	144	22	27	224×224	7
Inceptionv3 [28]	315	48	89	299×299	23.9
ResNet18 [30]	71	18	44	224×224	11.7
ResNet50 [30]	177	50	96	224×224	25.6
ResNet101 [30]	347	101	167	224×224	44.6
InceptionResv2 [29]	824	164	209	299×299	55.9
Xception [32]	170	71	85	299×299	22.9
DenseNet201 [31]	708	201	77	224×224	20
MobileNetv2 [33]	154	53	13	224×224	3.5
ShuffleNet [35]	172	50	5.4	224×224	1.4
NasnetMobile [34]	913	*	20	224×224	5.3
NasnetLarge [34]	1243	*	332	331×331	88.9
DarkNet19 [36]	64	19	78	256×256	20.8
DarkNet53 [37]	184	53	155	256×256	41.6
EfficientNetB0 [38]	290	82	20	224×224	5.3
SqueezeNet [40]	68	18	5.2	227×227	1.24

**Table 2:** The evaluation performance of the 19 CNN models describing the mean value (m) ± standard deviation (s) of a specific criterion over the five folds.

Network	mACC ± sACC	mSen ± sSen	mSpe ± sSpe	mF1 ± sF1	mFNR ± sFNR	mFPR ± sFPR	mPre ± sPre	mMathew ± sMathew	mErr ± sErr
AlexNet [24]	87.07 ± 5.11	84.9 ± 10.95	89.2 ± 3.7	86.39 ± 6.28	15.1 ± 10.95	10.8 ± 3.7	88.57 ± 3.49	74.6 ± 9.87	12.93 ± 5.11
Vgg16 [26]	89.7 ± 6.23	86.94 ± 9.34	92.4 ± 6.5	89.18 ± 6.9	13.06 ± 9.34	7.6 ± 6.5	91.98 ± 6.4	79.76 ± 12.1	10.3 ± 6.23
Vgg19 [26]	87.37 ± 7.01	83.27 ± 11	91.4 ± 10.33	86.58 ± 7.76	16.73 ± 11	8.6 ± 10.33	91.29 ± 9.02	75.64 ± 13.38	12.63 ± 7.01
GoogleNet (Inceptionv1) [27]	87.78 ± 5.87	87.55 ± 8.88	88 ± 11	87.65 ± 5.92	12.45 ± 8.88	12 ± 11	88.71 ± 9.11	76.3 ± 11.22	12.22 ± 5.87
Inceptionv3 [28]	92.93 ± 8.01	88.98 ± 11.82	96.8 ± 4.32	92.29 ± 9.05	11.02 ± 11.82	3.2 ± 4.32	96.11 ± 5.49	86.18 ± 15.55	7.07 ± 8.01
ResNet18 [30]	90 ± 5.68	89.18 ± 4.71	90.8 ± 10.13	89.97 ± 5.38	10.82 ± 4.71	9.2 ± 10.13	91.23 ± 9.32	80.41 ± 11.34	10 ± 5.68
ResNet50 [30]	92.42 ± 7.07	90.2 ± 11.24	94.6 ± 5.22	91.95 ± 7.81	9.8 ± 11.24	5.4 ± 5.22	94.2 ± 5.69	85.21 ± 13.85	7.58 ± 7.07
ResNet101 [30]	94.34 ± 7.28	92.86 ± 12.14	95.8 ± 3.19	93.89 ± 8.26	7.14 ± 12.14	4.2 ± 3.19	95.36 ± 3.94	88.96 ± 14.02	5.66 ± 7.28
InceptionResv2 [29]	90.3 ± 7.96	88.57 ± 10.54	92 ± 5.79	89.87 ± 8.63	11.43 ± 10.54	8 ± 5.79	91.34 ± 6.77	80.71 ± 15.82	9.7 ± 7.96
Xception [32]	88.99 ± 6.79	90 ± 7.85	88 ± 8.8	89.02 ± 6.68	10 ± 7.85	12 ± 8.8	88.39 ± 8.06	78.3 ± 13.59	11.01 ± 6.79
DenseNet201 [31]	93.94 ± 4.97	93.47 ± 3.86	94.4 ± 8.73	93.96 ± 4.7	6.53 ± 3.86	5.6 ± 8.73	94.75 ± 7.64	88.15 ± 9.6	6.06 ± 4.97
MobileNetv2 [33]	90.81 ± 7.24	85.51 ± 11.95	96 ± 3.39	89.9 ± 8.14	14.49 ± 11.95	4 ± 3.39	95.23 ± 4.32	82.25 ± 13.98	9.19 ± 7.24
ShuffleNet [35]	86.06 ± 6.84	80.61 ± 9.16	91.4 ± 14.33	85.24 ± 6.46	19.39 ± 9.16	8.6 ± 14.33	91.99 ± 11.52	73.6 ± 13.19	13.94 ± 6.84
NasnetMobile [34]	86.57 ± 6.47	80.82 ± 12.71	92.2 ± 5.97	85.25 ± 7.87	19.18 ± 12.71	7.8 ± 5.97	91.28 ± 5.36	74.09 ± 12.12	13.43 ± 6.47
NasnetLarge [34]	91.31 ± 7.08	88.16 ± 7.24	94.4 ± 7.7	90.96 ± 7.22	11.84 ± 7.24	5.6 ± 7.7	94.04 ± 7.9	82.84 ± 14.17	8.69 ± 7.08
DarkNet19 [36]	86.77 ± 4.14	81.02 ± 5.43	92.4 ± 3.36	85.79 ± 4.65	18.98 ± 5.43	7.6 ± 3.36	91.22 ± 3.95	73.98 ± 8.15	13.23 ± 4.14
DarkNet53 [37]	89.19 ± 6.15	83.88 ± 9.88	94.4 ± 2.97	88.26 ± 7.22	16.12 ± 9.88	5.6 ± 2.97	93.42 ± 4	78.87 ± 11.79	10.81 ± 6.15
EfficientNetB0 [38]	86.87 ± 3.44	85.31 ± 3.86	88.4 ± 4.88	86.56 ± 3.51	14.69 ± 3.86	11.6 ± 4.88	87.96 ± 4.91	73.86 ± 7.03	13.13 ± 3.44
SqueezeNet [40]	84.65 ± 2.38	86.73 ± 4.95	82.6 ± 6.19	84.83 ± 2.31	13.27 ± 4.95	17.4 ± 6.19	83.34 ± 4.8	69.66 ± 4.75	15.35 ± 2.38

**Table 3:** The obtained accuracies over five folds in the 19 CNN models.

Model	Fold1	Fold2	Fold3	Fold4	Fold5
AlexNet	78.28	89.9	86.87	90.4	89.9
Vgg16	82.32	86.87	86.87	95.96	96.46
Vgg19	79.8	80.81	90.4	89.39	96.46
Inceptionv1	82.32	84.85	83.84	91.92	95.96
Inceptionv3	79.8	90.91	96.97	99.49	97.47
ResNet18	82.83	85.35	92.93	92.42	96.46
ResNet50	81.31	90.91	92.93	97.98	98.99
ResNet101	81.82	94.44	96.97	98.99	99.49
InceptionResv2	77.27	88.89	93.43	93.94	97.98
Xception	78.28	86.87	90.91	93.43	95.45
DenseNet201	86.36	91.94	96.46	97.98	97.47
MobileNetv2	81.31	86.87	89.9	97.47	98.48
ShuffleNet	77.27	83.84	84.85	88.38	95.96
NasnetMobile	78.28	86.36	85.86	85.86	96.46
NasnetLarge	80.3	88.89	92.93	96.46	97.98
DarkNet19	81.31	85.86	84.85	90.91	90.91
DarkNet53	79.29	87.37	91.41	93.94	93.94
EfficientNetB0	84.34	83.84	85.35	88.89	91.92
SqueezeNet	82.32	84.85	82.32	85.86	87.88

**Table 4:** Normalized decision matrix. Alter.—alternative; Cr.—criterion.

Alter./ Cr.	ACC	Sen	Spe	F1-Score	FNR	FPR	Pre	MCC	Err	Para
**AlexNet**	0.9229	0.9083	0.9215	0.9194	0.4325	0.2963	0.9215	0.8386	0.4377	0.0203
**Vgg16**	0.9508	0.9301	0.9545	0.9491	0.5000	0.4211	0.9570	0.8966	0.5495	0.0090
**Vgg19**	0.9261	0.8909	0.9442	0.9215	0.3903	0.3721	0.9498	0.8503	0.4481	0.0086
**Inceptionv1**	0.9305	0.9367	0.9091	0.9328	0.5245	0.2667	0.9230	0.8577	0.4632	0.1771
**Inceptionv3**	0.9851	0.9520	1.0000	0.9822	0.5926	1.0000	1.0000	0.9688	0.8006	0.0519
**ResNet18**	0.9540	0.9541	0.9380	0.9575	0.6035	0.3478	0.9492	0.9039	0.5660	0.1060
**ResNet50**	0.9796	0.9650	0.9773	0.9786	0.6663	0.5926	0.9801	0.9578	0.7467	0.0484
**ResNet101**	1.0000	0.9935	0.9897	0.9993	0.9146	0.7619	0.9922	1.0000	1.0000	0.0278
**InceptionResv2**	0.9572	0.9476	0.9504	0.9565	0.5713	0.4000	0.9504	0.9073	0.5835	0.0222
**Xception**	0.9433	0.9629	0.9091	0.9474	0.6530	0.2667	0.9197	0.8802	0.5141	0.0541
**DenseNet201**	0.9958	1.0000	0.9752	1.0000	1.0000	0.5714	0.9858	0.9909	0.9340	0.0620
**MobileNetv2**	0.9626	0.9148	0.9917	0.9568	0.4507	0.8000	0.9908	0.9246	0.6159	0.3543
**ShuffleNet**	0.9122	0.8624	0.9442	0.9072	0.3368	0.3721	0.9571	0.8273	0.4060	0.8857
**NasnetMobile**	0.9176	0.8647	0.9525	0.9073	0.3405	0.4103	0.9497	0.8328	0.4214	0.2340
**NasnetLarge**	0.9679	0.9432	0.9752	0.9681	0.5515	0.5714	0.9785	0.9312	0.6513	0.0139
**DarkNet19**	0.9198	0.8668	0.9545	0.9130	0.3440	0.4211	0.9491	0.8316	0.4278	0.0596
**DarkNet53**	0.9454	0.8974	0.9752	0.9393	0.4051	0.5714	0.9720	0.8866	0.5236	0.0298
**EfficientNetB0**	0.9208	0.9127	0.9132	0.9212	0.4445	0.2759	0.9152	0.8303	0.4311	0.2340
**SqueezeNet**	0.8973	0.9279	0.8533	0.9028	0.4921	0.1839	0.8671	0.7830	0.3687	1.0000

**Table 5:** Ranking for decision making represented by the values of ϕ in PROMETHEE and *Q* in VIKOR. The highest ϕ value is the best, whereas the lowest *Q* is the best.

Model	ϕ: PROMETHEE	*Q*: VIKOR	PROMETHEE	VIKOR
AlexNet	−86.54004365	0.78423285	15	15
Vgg16	16.31877628	0.51048488	8	9
Vgg19	−63.8124359	0.74766659	13	13
Inceptionv1	−57.19966687	0.68096691	12	12
Inceptionv3	132.2050634	0.18466346	3	3
ResNet18	15.25546934	0.4614654	9	8
ResNet50	115.1633097	0.21251132	4	4
ResNet101	150.8418215	0	1	1
InceptionResv2	28.425464	0.4496109	7	7
Xception	−29.98203689	0.60787425	11	11
DenseNet201	133.2355605	0.07998389	2	2
MobileNetv2	72.89230795	0.42167181	6	6
ShuffleNet	−106.9819714	0.8594925	18	18
NasnetMobile	−89.20093646	0.84854	16	17
NasnetLarge	73.3193101	0.33461685	5	5
DarkNet19	−76.30565263	0.81073772	14	16
DarkNet53	1.456682009	0.56957337	10	10
EfficientNetB0	−95.9301979	0.78239429	17	14
SqueezeNet	−133.1608231	1	19	19

**Table 6:** Optimal CNN model selection in PROMETHEE versus VIKOR approach.

Model Rank	PROPMETHEE	VIKOR
1	ResNet101	ResNet101
2	DenseNet201	DenseNet201
3	Inceptionv3	Inceptionv3
4	ResNet50	ResNet50
5	NasnetLarge	NasnetLarge
6	MobileNetv2	MobileNetv2
7	InceptionResv2	InceptionResv2
8	Vgg16	ResNet18
9	ResNet18	Vgg16
10	DarkNet53	DarkNet53
11	Xception	Xception
12	Inceptionv1	Inceptionv1
13	Vgg19	Vgg19
14	DarkNet19	EfficientNetB0
15	AlexNet	AlexNet
16	NasnetMobile	DarkNet19
17	EfficientNetB0	NasnetMobile
18	ShuffleNet	ShuffleNet
19	SqueezeNet	SqueezeNet

## Data Availability

A publicly available dataset was analyzed in this study. This data can be found in https://challenge.isic-archive.com/data, accessed on 18 May 2021. Both the data analyzed during the current study and code are available from the corresponding author upon request.

## Abbreviations

The following abbreviations are used in this manuscript:

DL	Deep Learning
CNN	Convolutional Neural Networks
M	Malignant
B	Benign
CAD	Computer-Aided Diagnosis
FPR	False Positive Rate
FNR	False Negative Rate
MCDM	Multi-Criteria Decision Making
PROMETHEE	Preference Ranking Organization Method for Enrichment of Evaluations
VIKOR	Visekriterijumska Optimizacija I Kompromisno Resenje
